# The Importance of Developing Electrochemical Sensors Based on Molecularly Imprinted Polymers for a Rapid Detection of Antioxidants

**DOI:** 10.3390/antiox10030382

**Published:** 2021-03-04

**Authors:** Marie Elhachem, Philippe Cayot, Maher Abboud, Nicolas Louka, Richard G. Maroun, Elias Bou-Maroun

**Affiliations:** 1 UMR PAM A 02.102, Procédés Alimentaires et Microbiologiques, University Bourgogne Franche-Comté, AgroSup Dijon, 1 Esplanade Erasme, 21000 Dijon, France; marie.el-hachem@agrosupdijon.fr (M.E.); philippe.cayot@agrosupdijon.fr (P.C.); 2 Centre d’Analyses et de Recherche, Laboratoire CTA, UR TVA, Faculty of Sciences, Saint Joseph University, Beirut 1104 2020, Lebanon; nicolas.louka@usj.edu.lb (N.L.); richard.maroun@usj.edu.lb (R.G.M.); 3 UEGP Unité Environnement, Génomique et Protéonique, Faculty of Sciences, Saint Joseph University, BP 17-5208 Mar Mikhael, Beirut 1104 2020, Lebanon; maher.abboud@usj.edu.lb

**Keywords:** antioxidants, cyclic voltammetry, differential pulse voltammetry, screen printed electrodes, molecular imprinting, radical polymerization, sol-gel technique, electrochemical sensor, industrial applications

## Abstract

This review aims to pin out the importance of developing a technique for rapid detection of antioxidants, based on molecular imprinting techniques. It covers three major areas that have made great progress over the years in the field of research, namely: antioxidants characterization, molecular imprinting and electrochemistry, alone or combined. It also reveals the importance of bringing these three areas together for a good evaluation of antioxidants in a simple or complex medium, based on selectivity and specificity. Although numerous studies have associated antioxidants with molecular imprinting, or antioxidants with electrochemistry, but even electrochemistry with molecular imprinting to valorize different compounds, the growing prominence of antioxidants in the food, medical, and paramedical sectors deserves to combine the three areas, which may lead to innovative industrial applications with satisfactory results for both manufacturers and consumers.

## 1. Introduction

The world of antioxidants has always interested researchers because of its importance in many sectors. Their main role is to protect against oxidation caused by free radicals, through different mechanisms of action, presented in the following [1]. Antioxidants are very numerous, they exist in both natural and synthetic forms and there are many conventional and unconventional methods developed for their extraction, detection and for the evaluation of the antioxidant capacity they provide, but each has its advantages and disadvantages [2,3]. Electrochemistry, voltammetry in particular, is suggested as a solution capable of overcoming the obstacles imposed by these techniques, they are based on fast, easy, and very affordable techniques. In voltammetry, the current is measured by varying the potential applied to the electrode. Cyclic voltammetry (CV) and differential pulse voltammetry (DPV) are discussed in this review, being the most commonly used in the determination of antioxidants [4]. A large number of applications of these techniques have been carried out, with antioxidant and other compounds, and in several complex matrices. In addition, screen printed electrodes have been able to replace conventional electrodes, reducing the volume of solvents used and eliminating the problem of cleaning and reproducibility of the electrode [5,6]. A good application depends on a good choice of factors. The type of electrode, the solvent, the supporting electrolytes, the method parameters, and many others, contribute significantly to the efficiency of the technique.

In order to optimize selectivity, an innovative technique can be added to the antioxidant-electrochemistry couple, which can be used to transfer a signal to a specific antioxidant or family of antioxidants, which is molecular imprinting. It is a technique that consists of creating complementary images in terms of structure and functionality to a target molecule. This happens by creating within a synthetic polymer recognition sites specific to this molecule, in order to enhance its selectivity in the medium. There are different techniques and approaches used for molecular imprinting: radical polymerization and sol-gel process, where molecularly imprinted polymers (MIPs) and molecularly imprinted silica (MIS) are respectively obtained. Each has a large selection of reagents, the most important factor is to choose the appropriate reagents and conditions required for polymer synthesis. At the end of the synthesis, the washing step leaves cavities for recapturing the target molecule in a simple or complex medium [7]. MIPs and MIS have been extensively used in a wide range of applications, some of which are presented in this review. Their application with antioxidants for extraction, quantification, or purification purposes is large, as well as with electrochemistry alone to capture other molecules. 

This review highlights the importance of combining these three fields: antioxidants, electrochemistry, and molecular imprinting, in order to create a rapid and specific antioxidant detection method, involving the use of electrochemistry and molecular imprinting.

## 2. Antioxidants

Antioxidants, a subject that has always been a major attraction to the world of research, exist in natural and synthetic forms. However, since the consumer has become more concerned regarding his safety, the importance is being attributed to naturally occurring antioxidants in foods. 

Natural antioxidants are widely used to protect oxidizable species commonly found in pharmaceuticals, paramedical products, cosmetics, and foods. They were first used as food preservative, to extend the shelf life of food products and preserve their nutritional and organoleptic qualities. In addition, they protect human metabolism and prevent many health diseases such as colon and breast cancer, cardiovascular diseases, neurodegenerative diseases, chronic inflammatory diseases, osteoporosis, and others as a result of their anti-proliferative, pro-apoptotic, anti-inflammatory, neuroprotective, neuromodulator, antiviral, and many other effects [8,9,10,11,12,13].

The main targets of antioxidants are reactive oxygen species (ROS), such as free radicals mainly derived from oxidation reactions that target different structures (lipids, proteins, and carbohydrates) and that can affect foods and health [13].

### 2.1. Common Types of Food Oxidation

**Figure 1:**
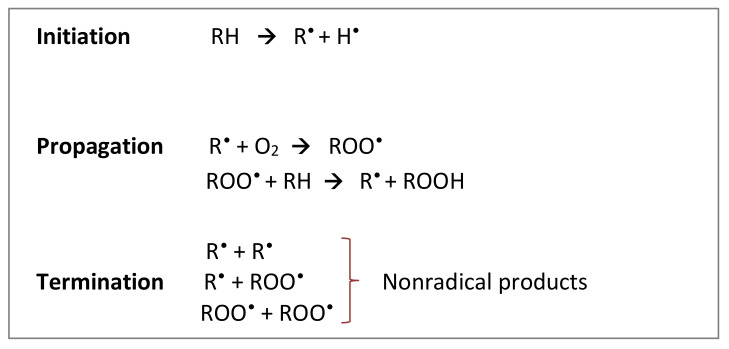
The general process of lipid peroxidation [14]. RH: target polyunsaturated fatty acid; R^•^: fatty acid radical; ROO^•^: fatty acid peroxyl radical; ROOH: lipid hydroperoxides

Lipid peroxidation is a very common type of oxidation that occurs in foods rich in unsaturated fatty acids and cholesterol. The free radical mechanism of lipid oxidation is usually divided into three stages: initiation, propagation, and termination (Figure 1) [14]. In the initiation stage, different factors can lead to free radical formation, such as temperature, light, heavy metals, or other free radicals. During the propagation stage, lipid radicals react with oxygen to form peroxyl radicals. Formed at this stage, peroxyl radicals react with another molecule of lipid, forming a lipid radical and a hydroperoxide that is not stable and decomposes easily to form primary then secondary products. All these products affect the quality and the taste of the food product. Secondary products are responsible for off-flavor. The latter is one of the main causes of oxidized food rejection by consumers. During the termination stage, radicals react with each other and form nonradical products. Any reaction that prevents the propagation of peroxidation or removes free radicals from the system plays a key role in the termination mechanism. This is where the importance of antioxidants comes in.

Similarly to lipid peroxidation, protein oxidation has an important impact on food quality, although it is less explored. 

Protein oxidation mainly depends on its amino acids composition or the primary structure, and thus their chemical structure. Table 1 shows the most reactive amino acids and their oxidation products. There are sulfur-containing amino acids, such as cysteine, that once oxidized, leads to thiyl radicals (RS**^•^**) and then generates a thiylperoxyl radical (RSOO**^•^**) or disulfide bond (RSSR) [15] according to the following reactions: 

RSH + ^•^OH → RS^•^ + H_2_O

RS^•^ + O_2_ → RSOO^•^

RS^•^ + RS^•^ → RSSR

**Figure 2:**
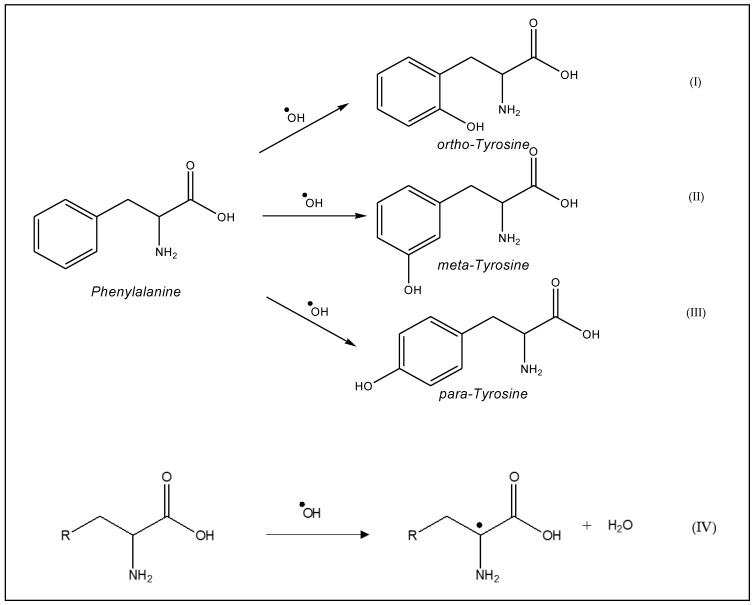
Oxidation process of: (**I**), (**II**), (**III**) aromatic amino acids and (**IV**) aliphatic amino acids [15].

Aromatic amino acids, such as tyrosine, tryptophan, histidine, and phenylalanine, are susceptible to oxygenation of their ring. For example, phenylalanine may generate *ortho*-tyrosine (Figure 2I) meta-tyrosine (Figure 2II), and 4-hydroxyphenylalanine (para-tyrosine) (Figure 2III) during oxidation. Aliphatic amino acids are oxidized by hydrogen abstraction at the alpha carbon and give a carbon-centered radical (Figure 2IV).

Besides controlling food processing conditions, feeding regime of the animal, and food storage, adding antioxidant to the product can also prevent food oxidation. Antioxidants may have close (ex: hydrophilic) or different (ex: lipophilic) effects on protein than those on lipids, and inhibition of protein oxidation can sometimes present a protective effect on the lipid fraction [16], and vice versa. Moreover, many proteins such as bovine serum albumin (BSA), β-lactoglobulin, lactoferrin [17,18,19], and protein hydrolysates from whey, casein, soy, and egg yolk [20,21,22,23] were found to have antioxidant effects themselves, by scavenging free radicals, chelating metals, reducing lipid hydroperoxides, and interacting with aldehydes [24].

### 2.2. Mechanism of Action

In general, the principle of antioxidants is based on avoiding radical initiation or propagation of radical state through several mechanisms. Antioxidants can be classified as primary or secondary antioxidants according to their mechanism of action. The primary or chain-breaking antioxidants (A:H in the following equations)) are able to give a hydrogen atom (Equation V) and a single electron (Equation VI) to a radical and thereby neutralizing it, such as phenolic compounds with one or more hydroxyl group (-OH). This mechanism is known for “radical scavenging,” although this term is not fully adapted to the reality of the mechanism. 

R^•^ + A:H → R^•^ + A^−^ + H^+^ → R^−^ + A^•^ + H^+^ → R:H + A^•^ (V)

R^•^ + A:H → R^−^ + A·H+ → R^−^ + H^+^ + A^•^ → R:H + A^•^ (VI)

**Figure 3:**
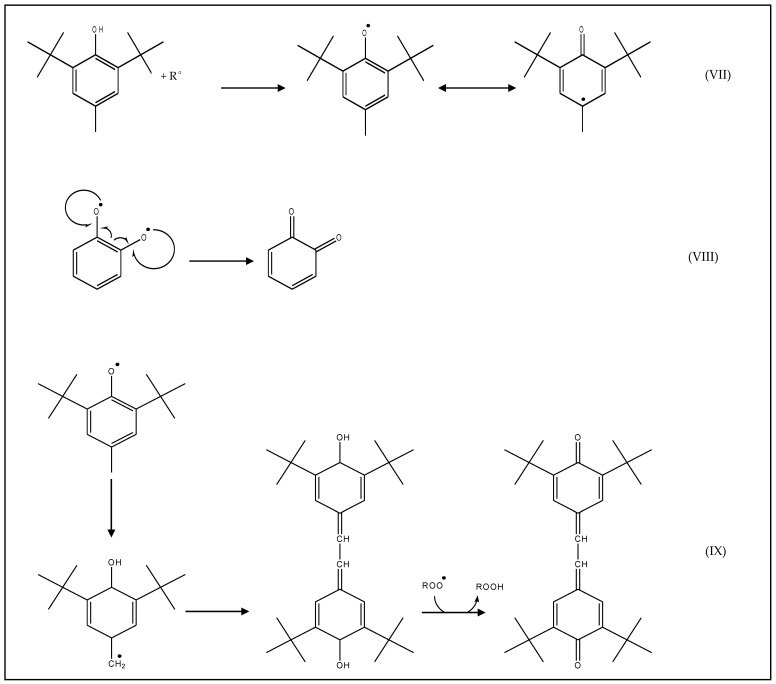
Example of stabilization by resonance, with (**VII**) butylated hydroxytoluene (BHT) and (**VIII**) ortho-diphenol, and example of BHT polymerization (**IX**).

Monophenols create unreactive phenoxyl radicals due to resonance stabilization (Figure 3VII), while diphenols, when oxidized, produce quinones (Figure 3VIII). Some of the monophenols (in a radical state and stabilized by resonance) can be polymerized, for example a natural antioxidant gamma-tocopherol or a synthetic antioxidant butylated hydroxytoluene (BHT), and give a non-radical dimer (Figure 3IX). These reactions disrupt the free radical chain propagation. 

**Figure 4:**
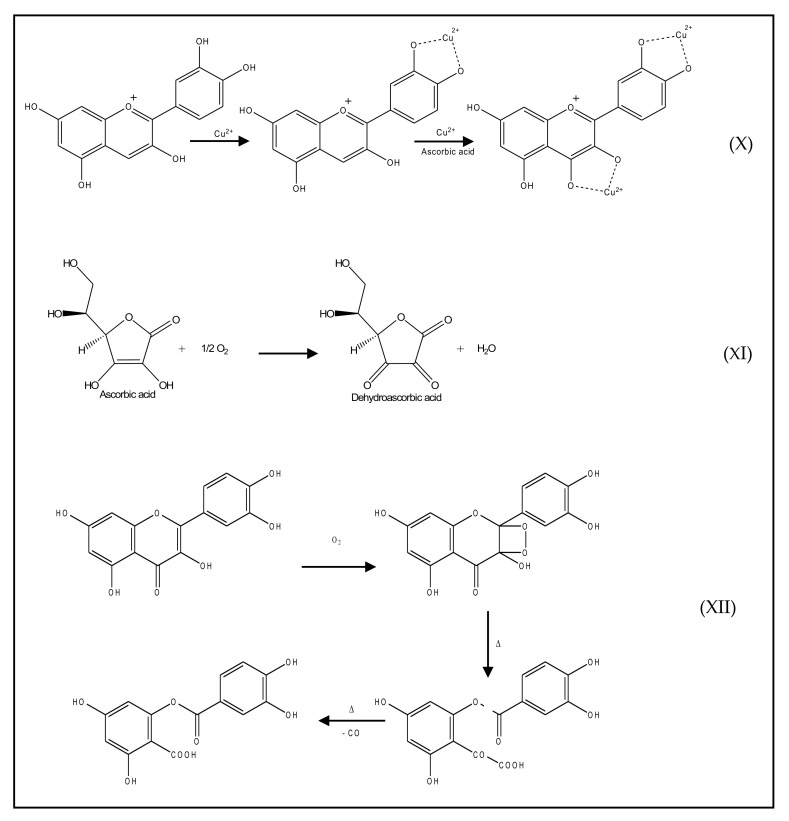
Reaction mechanisms of secondary antioxidants: (**X**) Metal ion (Cu^2+^) chelating activity of anthocyanidine (cyanidin), from [30] published by The Royal Society of Chemistry; (**XI**) oxygen scavenging activity of ascorbic acid, reproduced from [31] under Creative Common license; (**XII**) chemical reaction of quercetin with singlet oxygen, reproduced from [32] with the permission of Elsevier.

The secondary, or preventive antioxidants, are substances able to inhibit or delay chain initiation. Several mechanisms such as chelation of transition metals, oxygen scavenging, and quenching of singlet oxygen can be exhibited by these secondary antioxidants (Figure 4) [1,25,26,27,28,29].

### 2.3. Main Antioxidant Families

**Figure 5:**
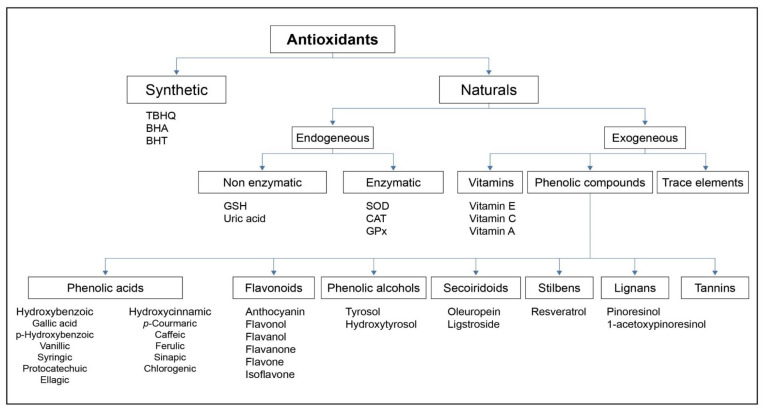
Antioxidant families. TBHQ: tert-Butylhydroquinone; BHA: butylated hydroxyanisole; BHT: butylated hydroxytoluene; GSH: reduced glutathione; SOD: superoxide dismutase; CAT: catalase; GPx: glutathione peroxidase.

Antioxidants are divided between endogenous and exogenous (Figure 5). One of the most interesting families of natural antioxidants is phenolic compounds. They are frequently found in food, such as anthocyanins and monomeric flavanols in red wine and berries, hydroxycinnamic and hydroxybenzoic acids in fruits and vegetables, ferulic acid in cereals, flavonols in tea, secoiridoids in olive oil, etc., [1,14,33,34,35,36,37,38,39,40,41].

### 2.4. Total Antioxidant Capacity Assays

Basically, to verify the efficiency of an antioxidant, the most convenient method is to compare a medium with and without added antioxidant and to record for several hours or weeks the content of the molecule of interest to be protected. For example, to evaluate the potential of an antioxidant for the preservation of fish oil, recording the oxidation of the oil (using peroxide value (PV), conjugated dienes (CD), thiobarbituric acid reacting substances (TBARS) methods) and comparing the blank (without antioxidants) and samples (oil added with different antioxidants at the same concentration) is the most accurate method to evaluate the efficiency of an antioxidant against the oxidation of the oil. The weakness of this strategy is the time required to obtain an evaluation of antioxidant efficiency.

**Figure 6:**
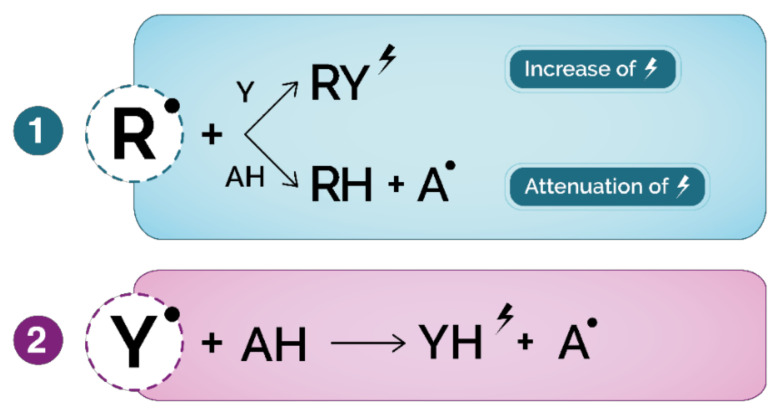
General mechanism of direct (competitive) and indirect (noncompetitive) antioxidant assays. (**1**) HAT-based assays: R^•^: free radical; Y: probe, AH: antioxidant; A^•^: oxidized antioxidant; 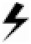: fluorescence, absorption, light emission, oxygen consumption; (**2**) ET-based assays: Y^•^: oxidized probe, AH: antioxidant; YH: reduced probe; 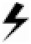: color modification of reduced probe.

Several methods have been used to apply rapid evaluation of the total antioxidant capacity (TAC) of complex samples (food extracts, beverages, biological fluids…), and they are classified into two categories according to their mechanism of action: hydrogen atom transfer (HAT) and electron transfer mechanisms (ET) (Figure 6).

The HAT-based assays are usually based on competitive reactions between the antioxidant and a suitable probe (oxygen, UV-Vis, fluorescent and chemiluminescent reagent) that reacts with the oxidant leading to changes in its measurable properties. The antioxidant capacity is calculated by measuring the fluorescence, absorbance, oxygen consumption or chemiluminescence decay curve of the probe and then integrating the area under the curve (AUC). These assays include total radical-trapping antioxidant parameter (TRAP), oxygen radical absorbance capacity (ORAC), and crocin-bleaching assays (CBAs) [30,32,40,41]. Oxygen radical absorbance capacity (ORAC) monitors the inhibition of peroxyl-radical induced oxidation by measuring the fluorescence decay of β-phycoerythrin or fluorescein as probe kinetically in the presence of antioxidants and an oxidizing agent generated by commonly used azo compounds such as lipophilic azo-bis(isobutyronitrile) (AIBN), 2,2′-azobis(2-amidinopropane) (ABAP), and 2,2′-azobis(2,4-dimethylvaleronitrile) (AMVN) or hydrophilic 2,2′-azobis(2-methylpropionamidine) dihydrochloride (AAPH). The greater the delay of fluorescence decay, the stronger the antioxidant capacity (AOC). In reality, ORAC is calculated using the area between the decay curve of blank and the curve with the sample containing an antioxidant. ORAC assay is supposed to measure lipophilic as well as hydrophilic antioxidants because it uses a mixed solvent of 50% acetone/50% water (*v*/*v*) containing 7% methylated beta-cyclodextrin to solubilize antioxidants [42].

Total radical-trapping antioxidant parameter (TRAP) monitors antioxidant capability to interfere with the reaction between oxygen or fluorescent (β-phycoerythrin) probe and ROO^•^ generated by an azo compound; and determines oxygen consumption or the fluorescence decay of β-phycoerythrin during oxidation inhibition. Ferric ion reducing antioxidant power (FRAP) and trolox equivalent antioxidant capacity (TEAC) are specific TRAP methods (see further in the ET-based assays) that use water solvent but there are other different FRAP techniques that use other solvents and probes, such as diphenyl-1-pyrenylphosphine (DPPP) or coumarin-triarylphosphin soluble in organic solvent [43] and could be adapted to evaluate liposoluble antioxidant. 

Crocin-bleaching assays (CBAs) monitors the inhibition of crocin-bleaching, based on competitive reaction of an antioxidant and UV-vis probe (crocin) with ROO^•^ generated by thermolysis of AAPH in the presence of O_2_; determines the absorption decay. This method concerns only water-soluble antioxidants (solvent: a 9:1 water-ethanol mixture) [44]. This method is not suitable to evaluate the radical scavenge capacity of liposoluble antioxidant.

Chemiluminescence measures antioxidant capacity in quenching several ROS, other than ROO^•^, such as hydrogen peroxide. It can be direct or indirect. It is based on the competition between antioxidants and chemiluminescent reagent (ex: luminol) for hydrogen peroxide; and monitors the decrease in light emission intensity caused by the antioxidant. Luminol is dissolved in aqueous borate buffer [45] with a small amount of ethanol to dissolve the standard, Trolox. This method is not suitable in evaluating the radical scavenge capacity of liposoluble antioxidant.

The ET-based assays are based on noncompetitive reactions, they measure the capacity of an antioxidant to reduce an oxidant probe and convert it to a colored, fluorescent or chemiluminescent species. The degree of color change or fluorescence decay is proportional to the antioxidant capacity. These assays include 2,2-diphenyl-1-picrylhydrazyl assay (DPPH), trolox equivalent antioxidant capacity (TEAC), ferric ion reducing antioxidant power (FRAP), cupric ion reducing antioxidant capacity (CUPRAC) assays, and the Folin-Ciocalteu assay [30,34,40,41]. Not to mention that some assays, such as DPPH and TEAC can be considered as mixed-mode methods (ET and HAT) because their radicals may be deactivated via HAT mechanisms, but studies showed better results via ET mechanisms [2,46,47,48,49]. 

Inhibition of 2,2-diphenyl-1-picrylhydracyl radical (DPPH^•^), a colorimetric method based on the capacity of antioxidants to neutralize DPPH^•^ radical, accompanied with absorbance decrease at 517 nm due to its decolorization is widely used as an indicator of the antioxidant efficacy. The DPPH method is performed generally in methanol, ethanol, or alcohol. This method is not suitable to evaluate the radical scavenge capacity of liposoluble antioxidant. DPPH was also used with a non protic solvent, ethylacetate [50] and even in an aprotic apolar solvent, toluene [51], in order to evaluate AOC of lipophilic antioxidant.

Trolox equivalent antioxidant capacity (TEAC), a colorimetric method based on the capacity of antioxidants to reduce 2,2′-azino-bis(3-éthylbenzothiazoline-6-sulphonique) (ABTS^•+^) radical, accompanied with absorbance decrease at maximum 734 nm, due to its decolorization. ABTS is soluble in water and is used for example for AOC assays with molecules extracted from plant with supercritical water [52]. This method is not suitable to evaluate the radical scavenge capacity of liposoluble antioxidant.

Ferric-reducing antioxidant power (FRAP), a colorimetric method based on the reduction of ferric ion Fe^3+^-tripyridyltriazine complex (Fe^3+^-TPTZ) to its blue colored ferrous form (Fe^2+^-TPTZ) by antioxidants, accompanied with absorbance increase at 593 nm. FRAP is a method that works in aqueous buffer [52]. This method is not suitable to evaluate the radical scavenge capacity of liposoluble antioxidant.

Cupric reducing antioxidant capacity (CUPRAC), similar to FRAP, is a method based on the reduction of cupric ion Cu^2+^-neocuproine (Cu^2+^-Nc) to cuprous ion (Cu^+^-Nc) by antioxidants, accompanied with absorbance increase at maximum 450 nm. CUPRAC works in water, possibly with a small amount of ethanol [53]. This method is not suitable to evaluate the radical scavenge capacity of liposoluble antioxidant. 

Folin-Ciocalteu reducing capacity, a colorimetric method based on the reduction of the Folin-Ciocalteu reagent (phosphomolybdic/phosphotungstic acid complexes) to a blue-colored chromophore by phenolic compounds, with maximum absorption at 765 nm. This method uses aqueous buffer [52] or in 1:1 methanol-water buffer [54]. This method is not suitable to evaluate the radical scavenge capacity of liposoluble antioxidant.

Despite the evolution and development of these techniques throughout the years, and all the advantages that they had presented to research in the field of antioxidants, they nevertheless involve limitations and disadvantages that push researchers to seek alternatives in order to improve their studies. Most of them are costly, not sufficiently rapid, and present lack of specificity. Although some of the assays were adapted to measure lipophilic as well as hydrophilic antioxidants, they still present an irrelevant classification. Different explanations can be given. The chemical reaction mechanisms and kinetics do not mimic the mechanism of antioxidant in situ as protector of molecules of interests. All these methods require standardization because results can differ between reactions due to several factors, such as type and amount of solvent, pH, presence of metal ions and antioxidant reaction, even with the same sample. For example, hydrophobic antioxidants, soluble in oil and efficient to protect oil against its oxidation, cannot be dissolved in acetone or alcohol with DPPH methods. In addition, the same antioxidant evaluated by different assays or by the same assay in different laboratories may give rise to serious differences in results. Actually these differences in antioxidant capacity or rank lead to a lack of correlation between activities [30,55].

HAT-based methods suffer from different limitations. A common one is that antioxidant extracts may naturally contain pigments and fluorophores that can interfere with absorbance and fluorescence affecting the results. Moreover, β-phycoerythrin probe used in ORAC and TRAP can interact with phenolic compounds by nonspecific protein binding and cause underestimation of antioxidant capacity, not to mention that its reactivity toward peroxyl radicals can vary each time. When oxygen is used as a detection probe, it can affect the results because of its instability. Oxygen pressure cannot be controlled, which makes it impossible to control the peroxide content. In addition to that, many methods require automated systems that cannot be found in all laboratories, same for reagents not easily available commercially, such as crocin. On the other hand, these methods have a lag-phase that is not the same for all antioxidants, and they present ambiguity in end-point determination, which makes data comparison between laboratories more difficult [55].

ET-based methods have been criticized mainly because they ignore the reaction kinetics, and many probes used as oxidant are non-physiological radicals (ABTS^•+^ and DPPH^•^) which makes the results incomparable to the real-life antioxidant action [2,46,47,48,55].

### 2.5. Extraction and Detection of Antioxidants

A wide range of analytical methods were developed for the extraction of antioxidants from food and their by-products, conventional (Soxhlet extraction, liquid–liquid extraction, solid phase extraction) and non-conventional or emergent techniques (ultrasound, microwave, pulsed electric fields (PEFs), high-voltage electrical discharges, ultrasounds, infrared, supercritical and subcritical fluid extraction, instant controlled pressure drop (DIC), and intensification of vaporization by decompression to the vacuum (IVDV)), and for their content detection (HPLC with UV, fluorescence or photodiode array detector, thin layer chromatography, capillary electrophoresis, supercritical fluid chromatography) [56,57]. However, there are several limitations in using most of them. For example, conventional extraction techniques are time, solvent, and energy consuming, in addition to the fact that antioxidants are strongly influenced by many important parameters such as the type of solvent used, pH, temperature, etc., [58,59,60,61]. Non-conventional techniques presented serious advantages but they also hold some disadvantages that should not be underestimated when choosing the technique. Ultrasound and pressure assisted extractions are expensive, microwave-assisted extraction involves quick heating, which risks burning the sample and breakdown of antioxidant compounds, not to mention that solubility should be considered [62]. Moreover, further studies are needed to highlight the energy consumption of these technologies and their environmental impact.

Moving on to detection techniques that are time consuming, they require sample preparation and pre-treatment. Moreover, interfering substances affect the extract purity, and the presence of structurally similar compounds to antioxidants that belong to the same of different families makes selective extraction difficult where many components can be determined simultaneously. 

To avoid these problems or at least attenuate them, researchers attributed a special interest to electrochemistry, an alternative method that has been widely used and developed due to its ability to overcome all the obstacles mentioned above in order to enhance the evaluation of antioxidant activities [63,64,65].

## 3. Electrochemistry

Why electrochemistry? Electrochemistry has numerous advantages. It is fast, sensitive enough for physiological determinations of antioxidants at low limits, affordable, easily accessible in the market. Also it involves simple analytical procedures that does not require complicated and time-consuming sample pre-treatment or any addition of reactive species, etc. Additionally, electrochemical methods may determine several parameters that help understand antioxidants’ reaction mechanism, such as redox potential, electrons number, quantity of electric charge, etc. 

Electrochemistry works usually with aqueous but can use non-aqueous electrolyte solutions, with a large range of solvent having a high value of permittivity (e.g., formamide, ε = 111, µ = 3.73 D) but also with low permittivity value and a low dipole moment (e.g., 1,4-dioxane, ε = 2.3, μ = 0.45D). Except with full apolar solvent (dipolar moment close to zero, µ → 0) and very viscous solvent, it is possible to use solvent suitable for lipophilic antioxidant, using surfactant [66] or complex support electrolytes, such as tetrabutylammonium hexafluorophosphate (TBAPF6), dissolved in a mixture of organic solvents, dichloromethane and acetonitrile [67], or in acetonitrile alone [68].

Electrochemistry has given rise to several electroanalytical methods that have grown greatly in application and importance to offer high sensitivity and precision and allow quantitative evaluations to be made on a variety of samples with relatively low-cost instrumentations. These methods are classified based on the measured signal: (1) Amperometry measures the current resulting from a constant potential at different times; (2) voltammetry, a subclass of amperometry, measures the current by varying the potential applied to the electrode; and (3) potentiometry measures the potential of a solution between two electrodes. Electrochemical methods showed viable results in many applications such as food, clinical, and pharmaceutical analysis [4].

These electroanalytical techniques, especially voltammetry, have received a special interest in the world of natural antioxidants that are usually known to be electroactive or redox active compounds. The performance of voltametric techniques is highly influenced by the material of the working electrode. Glassy carbon electrode (GCE) is the most frequently used, but other commonly used materials are platinum, gold, silver, graphite, and carbon paste. The field of modified electrodes has been one of the most active areas of research interest with a large number of applications, where a thin film is coated on the surface of the electrode leading to changing the functionality of its material and enhancing its electronic and structural properties. However, GCE are costly, and require time-consuming preparation, not to mention the necessity to clean it prior to each measurement in order to obtain reproducible results. Cleaning the electrode is a critical step, as it could alter the performance of the electrode, as demonstrated by Lima et al. [69], where the cleaning of the GCE involves its polishing on alumina slurry, leaving alumina residues that affect the electrochemical parameters of the antioxidants. Alternatives have been proposed, in order to avoid these disadvantages. Pencil graphite electrodes (PGE) were fabricated; they are simple, disposable, cheap, and widely commercially available. Their electrochemical performance was well demonstrated [70,71,72,73]. Moreover, a very well established approach used for the development of electrochemical sensors is the screen-printed electrodes (SPE). They are small, fast, inexpensive, reliable, and easy to use. They allow performing a large number of experiments with small volumes of sample and the fact that they are single use sensors eliminates pre-treatment and maintenance procedures. They are versatile and customizable, a large variety of materials and configurations of working electrode are available, and even modified electrodes [5,6,63]. 

Cyclic voltammetry (CV) and differential pulse voltammetry (DPV), provided with several types of working electrodes, are among the most extensively used electroanalytical techniques for studying redox reactions and for evaluating qualitative and quantitative aspects of antioxidants [64,74,75,76,77,78,79,80,81]. Voltammograms profiles are determined by the variation of the current with the applied potential.

CV method is based on a controlled potential variation. CV voltammogram is usually represented by an electrochemically reversible reaction showing only one anodic peak (E_pa_) and one cathodic peak (E_pc_) resulting from the redox potential of the studied antioxidant in a specific medium, which provides information about the integrated antioxidant capacity. The more susceptible the compounds are to oxidation, or in other terms the greater their antioxidant capacity, the sooner they will reach the anodic peak potential [82]. Such reversible systems are generated by ortho-diphenols, whereas for quasi-reversible (moderate-sized cathodic peak) and irreversible systems (absence of cathodic peak), where electron transfer is progressively slower, the peaks are separated and reduced in size. A quantitative relationship exists between the reduction potential and concentration of the redox couple, according to Nernst Equation (1):

(1) $${E=E}^{0}+\;\frac{\mathrm{RT}}{\mathrm{nF}}\ln\left(\frac{\left[\mathrm{Ox}\right]}{\left[\mathrm{Red}\right]}\right)$$

where E^0^ is the formal reduction potential, R is the gas constant, T is the temperature, n is the number of electrons transferred in the redox event, F is Faraday’s constant, and [Ox] and [Red] are the interfacial concentrations of the oxidized and reduced species respectively [83].

The potential is measured between the working electrode and the reference electrode, while the current is measured between the working electrode and the counter electrode [84,85]. 

DPV method involves two measurements of the current for each potential pulse: before (I_1_) and at the end (I_2_) of the application of the pulse, which makes DPV techniques much more sensitive than CV (cyclic voltammetry) [74], and the difference (ΔI = I_2_ − I_1_) is plotted according to the potential applied. The voltammogram has a differential shape that presents a current peak, its height is directly proportional to the concentration of the studied antioxidant. The electrochemical cell is similar to that of CV, in which the potential is measured between the working electrode and the reference electrode and the current is measured between the working electrode and the counter electrode [63,85]. 

Some of the experiments using CV and DPV techniques for the determination of several compounds with different types of electrode materials are respectively listed in Table 2.

The choice of electrodes is a key point in electrochemical analysis, but the solvent used and the supporting electrolytes are also important in electrochemistry.

The solvent used in electro organic reactions must fulfill several specifications: (i) good solubility of the supporting electrolytes and substrates to the solvent, (ii) high electroconductivity, (iii) high electrochemical stability, and (iv) suitable chemical reactivity [100].

The supporting electrolytes have to be non-electroactive in the range of applied potentials [105,106], and they can be different according to the species analyzed and their oxidation and reduction potential: LiCl or KCl; tetrabutylammonium salts with different counter-ions such as acetate, benzoate, bromide, chloride, hexafluorophosphate, tetraphenylborate, tetrafluoroborate, perchlorate. The selection of supporting electrolytes should take into consideration several points: (i) solubility to the solvent used, (ii) electrochemical stability, (iii) interaction with reaction intermediate, and (iv) relative difficulty of preparation. 

**Figure 7:**
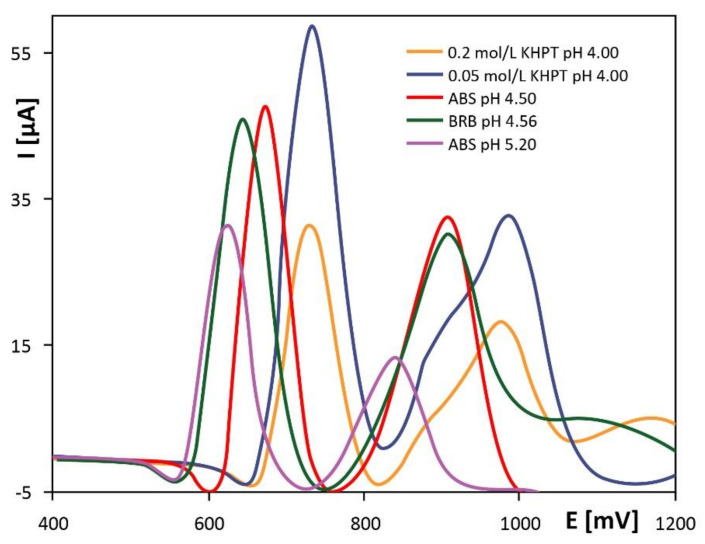
Differential pulse voltammograms recorded at PGE* for 6.00 × 10^−5^ mol/L NGN in different supporting electrolytes. Reproduced from [71] with the permission of Royal Society of Chemistry. NGN: naringenin, KHPT: potassium phthalate monobasic, ABS: acetate buffer solution, BRB: Britton–Robinson buffer.

A very specific and recent study used a disposable electroactivated PGE (PGE*) to investigate the electrochemical behavior of the flavonoid naringenin (NGN). In this study, several supporting electrolytes were tested with low pH values. Differential pulse voltammograms recorded at PGE for 6.00 × 10^−5^ mol/L NGN showed the highest signal of NGN oxidation peak obtained at 0.05 mol/L potassium phthalate monobasic (KHPT) pH 4.0 (Figure 7) [71].

Other studies proved several adequate solvent/electrolyte systems, such as: tetra-n-butyl ammonium tetrafluoroborate (BF_4_^−^) in dichloromethane for electrochemical evaluation of lipophilic antioxidants [107]; or tetrabutylammonium hexafluorophosphate (Bu_4_NPF_6_) in oxygen saturated acetonitrile solution [108] using glassy carbon electrode and CV. The combination of these solvents and support electrolyte system was used in different applications. For phenol acids or polyphenols, simple solutions have been used, in the following examples. A caffeic acid solution in sulfuric acid (H_2_SO_4_) was used for voltametric determination of caffeic acid in red wines, using nitrogen-doped carbon/glassy carbon electrode and DPV [109]. A simple phosphate buffer solution was preferred for the determination of gallic acid and total polyphenols in wine samples using carbon paste electrode modified with carbon nanotubes under differential pulse voltammetry conditions [95]. As a last example of diluted sulfuric acid solution as solvent, H_2_SO_4_ in 1:2 (*v/v*) benzene/ethanol have been used for electrochemical evaluation of tocopherols behavior, using solid platinum electrodes and pulse voltammetry, cyclic voltammetry, and linear sweep voltammetry [63].

Electroanalytical experiments applied for the determination of antioxidants concentrations and/or antioxidant capacity have led to reliable results with a large number of perspectives and suggestions in order to allow researchers improve their studies and better understand the activity of antioxidants and their effects in different media. However, this complementary couple, electrochemistry-antioxidants, also had some limitations, one of which is the lack of specificity.

Given the instability of antioxidants, their high reactivity, their abundance, and their structural similarity, it is often difficult to report the results of a specific antioxidant. One solution is a rapidly growing technique, which has yielded very promising results in term of specificity and selectivity, it is molecular imprinting technology. The use of electrode-containing molecularly imprinted polymers has improved antioxidant studies and enhanced the selectivity of the results [110,111].

## 4. Molecular Imprinting

### 4.1. MIP Synthesis and Applications

**Figure 8:**
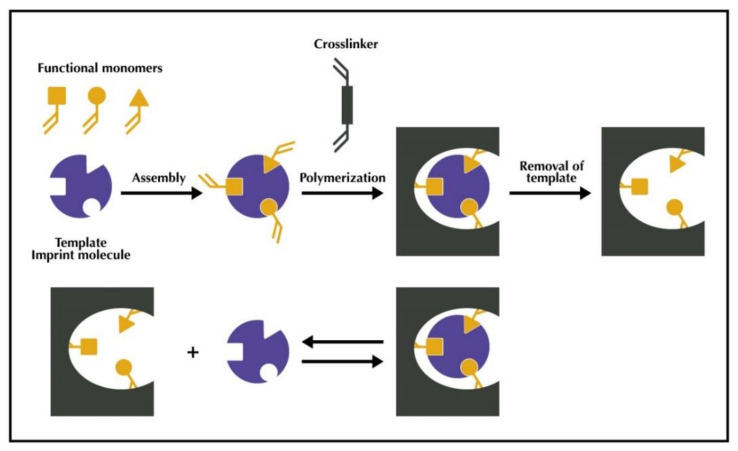
General principle of molecular imprinting.

Molecular imprinting technology is a technique that has been attracting the interest of the scientific community for more than 20 years, due to its simplicity, low cost, easy preparation, high selectivity and simplicity, resulting in a great increase in the literature in this field. This technique consists of creating complementary images in terms of structure and chemical functionalities of a target molecule within a synthetic polymer (Figure 8) by creating recognition sites within a polymer with a complementary geometrical and chemical fitting structure, which presents high affinity and selectivity toward the target molecule.

The synthesis of MIPs requires the following reagents: (1) the template or target molecule to be imprinted, (2) functional monomers, (3) cross-linking agent, (4) polymerization initiator, and (5) porogenic solvent. Several options of each reagent exist, their choice affects the resulting polymer selectivity and depends on their ability to interact with the functional groups of the target molecule, and the synthesis approach used (radical polymerization or sol-gel process). 

The main phases of the synthesis process are the following: (1) Complexation between functional monomers and template molecules, through different interactions (covalent, semi-covalent, or non-covalent) to form the pre-polymerization complex; (2) polymerization of the pre-polymerization complex with the cross-linkers and initiators under thermal or UV conditions. It involves the bulk, precipitation, suspension, core-shell emulsion, surface imprinting, and multi-step polymerizations, and finally, (3) removal of the template that will reveal a well-defined cavity in the polymer characterized by having a complementary structure to that of the target. 

A great majority of molecularly imprinted polymers (MIPs) have been synthesized by radical polymerization. In this type of polymerization, the template molecule chosen should be stable and must not participate in the reaction mechanism or inhibit polymerization. Therefore it is necessary to make sure that it only contains functional groups that are inert during polymerization [112], otherwise it would be necessary to look for alternative printing strategies, such as sol-gel process or the protection of the function responsible for the antioxidant effect. Functional monomers are directly responsible for the structure of the recognition site in the resulting polymer. Some typical monomers are methacrlylic acid, acrylic acid, itaconic acid, 2-(trifluoromethyl)acrylic acid, 4-vinylpyridine, acrylamide, methacrylamid, and 2-hydroxyethyl. Molecular modelling can be used for the selection of the functional monomer and for the evaluation of the stability of the pre-polymerization complex [113,114,115]. It can also be used to study the effect of the porogenic solvent on the selectivity of MIPs [116]. Porogenic solvent acts as pore-forming agent and primarily affects the imprinting efficiency, the most frequently used solvents are (toluene, dichloromethane, methanol, acetonitrile, etc.). The choice of the solvent depends on the solubility of the chosen reagents [117]. Cross-linkers control the morphology of the polymer, stabilize the binding site, and give the polymer its mechanical stability [112]. The most commonly used cross-linkers are divinylbenzene, ethylene glycol dimethacrylate, and trimethylolpropane trimethacrylate. In most cases, polymerization is initiated by thermal or UV radiation (radical initiation). Many initiators can be used as a source of radicals during radical polymerization. Generally, initiators of the azo compound (-N=N-) type are used. By the fact that their radical cleavage is easy, they are able to initiate a large number of monomers, in particular thermal initiation. The most commonly used is azo bis(isobutyronitrile) or 2,2′-azobis(2-methylpropionitrile) (known by the abbreviation AIBN, fairly soluble in water or toluene and especially very soluble in dichloromethane), mostly used for thermal initiation, and 2,2-dimethoxy-2-phenylacetophenone (DMPAP) is a commonly used photoinitiator.

Molecularly imprinted polymers (MIPs) have been developed and applied for several purposes. Initially, they were used to enhance the extraction selectivity of target analytes in solid-phase extraction techniques (SPE), so-called molecularly imprinted SPE (MISPE) as alternatives to immunosorbents (time-consuming and expensive technique) [118,119] and aptamers (availability of a limited number of sequences) [120,121,122] for their cheap, easy and rapid preparation, high thermal and chemical stability. The first application was carried out by Sellergren in 1994 [123] for the direct extraction of pentamidine from diluted human urine samples, as a drug used to treat AIDS-related pneumonia. MISPE allowed the detection or the clean-up of many target analytes (Sudan I [124], caffeine [125] from food matrices, 17β-estradiol from fishery samples [126], etc.). Moreover, it was recently applied to the trace analysis of pesticides [127,128], industrial contaminants [129,130], and drugs [131,132] from environmental waters, and natural products from food or plants [133,134,135,136,137,138]. MISPE is usually coupled with high performance liquid chromatography (HPLC) [118,119,139,140], and MIPs can even be integrated and used as the stationary phase in liquid chromatographic columns [139,140,141,142,143], capillary electrophoresis (CE), and electrochromatography (CEC) [144,145]. Then, notable attention has been directed to MIPs for sensing applications, where they are integrated with several transduction platforms in order to create a chemical or biochemical sensor. The adhesion of the MIP on the transducer is a major factor in the sensor response, and it was developed over time. The evaluation of binding properties has advanced from absorbance measurements [146] to HPLC [147]. This approach was first used with acoustic [148] and optical transducers [149], then with electrochemical sensors [150].

### 4.2. MIPs-Antioxidants

MIPs have allowed a huge number of studies to achieve results that helped researchers to move forward and seek out further perspectives. One of the most important domains is antioxidant detection where a wide range of polymers was developed and designed for their recognition. Several antioxidant components are known for their structural similarity within the same or different families, the fact that made molecular imprinting technique of great interest in order to discriminate them. Some of the most commonly used antioxidants with molecular imprinting applications are listed in Table 3.

Molecular imprinting has even been incorporated into biomedical applications of antioxidants, such as the preparation of a controlled drug delivery device for α-tocopherol oral supplementation, where polymers where synthesized using methacrylic acid (MAA) as functional monomer and ethylene glycol dimethacrylate (EGDMA) as cross-linker, they showed a sustained drug release capacity in gastrointestinal simulating fluids [151]. The same complex was used in the synthesis of a selective developed for tocopherol recognition, which has proved to be suitable for the separation and extraction of tocopherols from biological media [152]. 

Some applications require the template to be imprinted in order to recognize a structurally similar molecule. For example, a MIP has been synthesized using quercetin as template, 4-vinylpyridine as functional monomer, and ethylene glycol dimethacrylate as crosslinker and was successfully applied to the clean-up and preconcentration of catechins from several natural samples [153]. 

Caffeic acid is a very common antioxidant. A molecularly imprinted polymer monolithic stationary phase was prepared in the chromatographic column using caffeic acid as template, MAA and EGDMA as functional monomer and cross-linker, respectively, was successfully applied to the separation and purification of chlorogenic acid from Eucommia ulmodies leaves by absorbing the impurities that co-existed in the extract [143].

As previously mentioned, one of the essential steps to verify when initiating the synthesis of a MIP is the inert state of the template. However, antioxidants are active compounds susceptible to react with free radicals. To be on the safe side of these issues, a convenient alternative to acrylic-based MIP is the sol-gel molecularly imprinted silicas (MIS) [165,166,167].

### 4.3. MIS-Antioxidants

**Figure 9:**
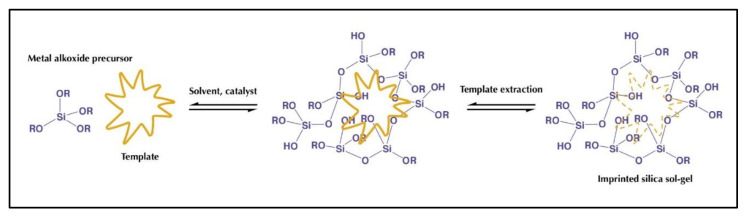
Hybrid organic-inorganic printing procedure.

The sol-gel process is based on two main steps: hydrolysis (acid or basic) and condensation. The most used functional monomers are alkoxysilane molecules, such as (3-Aminopropyl) triethoxysilane (APTES), (3-Aminopropyl) trimethoxysilane (PTMOS), N-[3-(Trimethoxysilyl)propyl] aniline (TMSPA), and phenyl trimethoxysilane. A very important point is that the solvent used is a hydroalcoholic solution, which means that, contrary to the organic solvents used in the synthesis of MIP, it respects the principles of green chemistry. Water:ethanol or water:methanol ratio varies according to the solubility of the template. The most commonly used cross-linker is tetraethoxysilane (TEOS) [166,168,169,170]. Figure 9 represents a hybrid organic-inorganic printing procedure.

MIS are more specific toward the target species than MIP, and they allow faster diffusion of analytes [166,171]. The silica-based materials allow the use of high temperature for template removal from the polymer network, a step that has always been challenging in MIP synthesis, and they provide products of high thermal and mechanical stability with longer lifetime [170,172]. L. Wang et al., (2019) [173] established a comparative study between three different molecularly imprinted polymers for gossypol, which showed that MIP prepared by bulk polymerization had a high adsorption capacity (564 mg/g) but MIS showed faster adsorption kinetics (40 min) [168]. 

Some of the applications of MIS in analytical and bioanalytical fields are solid-phase extraction of enrofloxacin from fish and chips samples [174], methyl-3-quinoxaline-2-carboxylic acid and quinoxaline-2-carboxylic acid from pork muscle [175], florfenicol from meat samples [176], polar organophosphorus pesticides from almond oil [177], iprodione fungicide from wine samples [167], methylxanthines from natural water and human urine [172], patulin from apple juice samples [178], vitamin D3 from aqueous samples [179], phenobarbital in human plasma [165], solid-phase microextraction of fentanyl [180] and bilirubin [181] from urine and plasma samples, etc.

As for antioxidants, MIS applications are not as extensive as those of MIPs, but they have however achieved satisfactory results. Many studies combined both approaches, they prepared acrylate-based MIPs followed by sol-gel process [182]. MIS monolith was developed in SPME for the separation and determination of gallic acid in orange juice samples [183], MIS microspheres were prepared for quercetin recognition [184], MIS mediated by aluminum ions was prepared for SPE of quercetin from Ginkgo biloba L. [185], carbon dots coated with MIS were successfully developed for caffeic acid detection [186], others were prepared for the recognition of caffeine [187,188].

## 5. Electrochemistry, Molecular Imprinting, and Antioxidants

Given the importance of electrochemistry, the usefulness of molecular imprinting, and the plethora of research on antioxidants, few studies have combined all three elements.

### 5.1. Electrochemistry and Antioxidants

Electrochemistry, especially cyclic voltammetry and differential pulse voltammetry, has been widely used in the detection of antioxidants, using conventional or screen-printed electrodes, with or without surface modification. For example, several studies used modified electrodes for caffeic acid determination in wine samples by electrochemical techniques, such as a (poly(3,4-ethylenedioxy)thiophene) modified electrode prepared using water-soluble polyelectrolyte poly(styrene-4-sulfonate) (PEDOT:PSS) [189], a screen printed carbon electrode modified with electrochemically reduced graphene oxide (ERGO/SPCE) [190], a nitrogen-doped carbon modified glassy carbon electrode (NDC/GCE). Other nanomaterial based approaches represented valid alternatives to conventional methods for polyphenols analysis (antioxidant capacity evaluation [93,95,191,192,193,194,195,196,197], total phenols estimation [198,199], o-diphenol evaluation [200,201], polyphenols studies [202,203,204], etc.). When compared to pulse techniques, cyclic voltammetry suffers from restricted limits of detection (10^−5^ M), and therefore from poor sensitivity and selectivity at the analysis of samples rich in antioxidants that are oxidized at potentials greater than 500 mV [205].

### 5.2. Electrochemistry and Molecular Imprinting

On the other hand, electrochemical biosensors based on molecularly imprinted polymers have been extensively designed for sensing applications of various biomolecules using modified electrodes, such as hormones [206,207,208,209], proteins [210,211,212,213,214,215], antibiotics [216,217,218,219,220,221,222], pesticides [223,224,225,226,227], neurotransmitters [228,229,230,231], etc.

One of the most challenging steps in the development of these sensors is the polymer deposition on the electrode, especially when screen-printed electrodes are used. Among these methods are dip coating, spin coating, drop casting, etc., where the polymer is prepared ex situ and then deposited on the surface of the electrode [232,233,234,235]. In addition, the synthesis can be performed in situ, by electropolymerization. It is a fast and straightforward means of obtaining polymer films on the surface of the electrode, by applying a range of potentials to a solution containing the pre-polymerization complex in presence of the template molecule. The advantages of this approach are the thickness control of the polymer obtained that influences the sensitivity of the imprinted electrochemical sensor, and the ability to attach the film to electrode surfaces of any shape or size [236,237,238,239].

Few electrochemical sensors based on molecular imprinting (MIP or MIS) for determination of antioxidants are developed.

**Figure 10:**
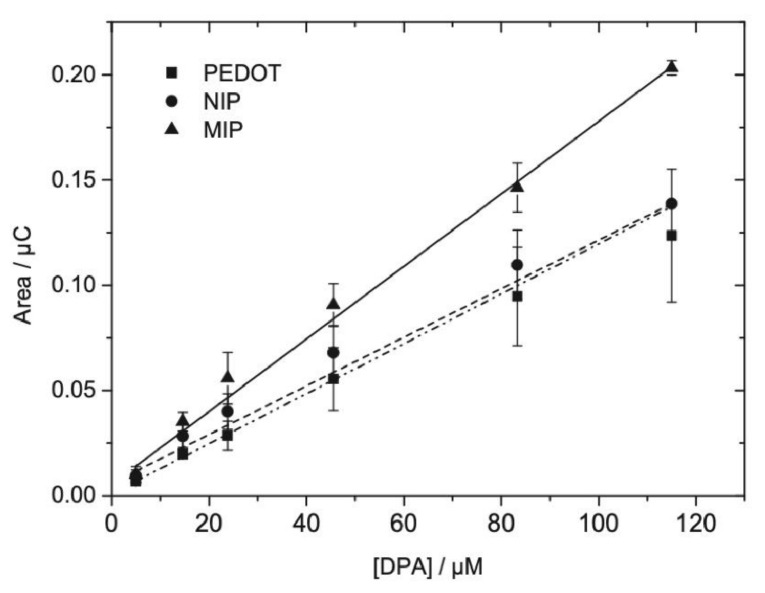
Calibration plots for the different sensors: with PEDOT film, with PEDOT/NIP membrane and with PEDOT/MIP membranes. Concentration range of 4.95–115 µM diphenylamine, reproduced from [240] with the permission of Elsevier.

A thin-film electrochemical sensor based on MIPs was prepared for diphenylamine detection. For MIP synthesis, MAA was used as functional monomer, trimethylolpropane trimethacrylate (TRIM) as cross-linker, 2,24-azobis(2-methylpropionitrile) as catalyst and acetonitrile as solvent. Microfabricated gold electrodes were cleaned, electrochemically activated, and well coated with electropolymerized poly(3,4-ethylenedioxythiophene) (PEDOT) membrane. The optimum membrane thickness of about 50 nm Then the prepared MIP was immobilized on the surface of the electrodes. Electrochemical responses of three electrodes with PEDOT membranes, containing the MIP, the NIP, and no particles (blank) have been investigated. Calibration of the three sensors showed that PEDOT/MIP electrodes displayed higher sensitivity compared to the electrodes with PEDOT and PEDOT/NIP (Figure 10). The response characteristics of PEDOT/MIP sensor were a sensitivity of 1.74 × 10^−3^ µC/µM in a linear range of 4.95–115 µM, a limit of detection of 5.4 µM, and a good selectivity in the presence of structurally similar compounds. The sensor was then applied to the quantification of diphenylamine in spiked apple juice samples [240].

A carbon entrapped molecularly imprinted polymer (CEMIP) electrode, made from scratch, was designed for electrochemical detection of resveratrol in wine using DPV, where carbon was tightly packed in a poly(MAA-co-EGDMA) polymer monolith fritted micropipette tip, then the MIP/NIP pre-polymer solution mixture, consisting of MAA as monomer, EGDMA as crosslinker, 4,40-azobis(4-cyanovaleric acid) (ACVA) as initiator, and acetonitrile as solvent, was infused on the carbon packed micropipette tip. A platinum wire was immersed, and the polymerization was initiated and kept overnight at 70 °C. The polymer was then washed and the CEMIP was exposed for chemical sensing. The CEMIP was 12 times more sensitive for the detection of resveratrol than the carbon entrapped non-imprinted polymer (CENIP). It had a detection limit of 20 µg/L with good linear standard addition calibrations with *R*^2^ = 0.99 for concentrations between 0.1 and 5 mg/L. Compared to the conventional carbon MIP composite electrode, the CEMIP was found to be more sensitive due to the accessibility of the resveratrol cavities with a more efficient electron transfer due to their thin layer design [241].

**Figure 11:**
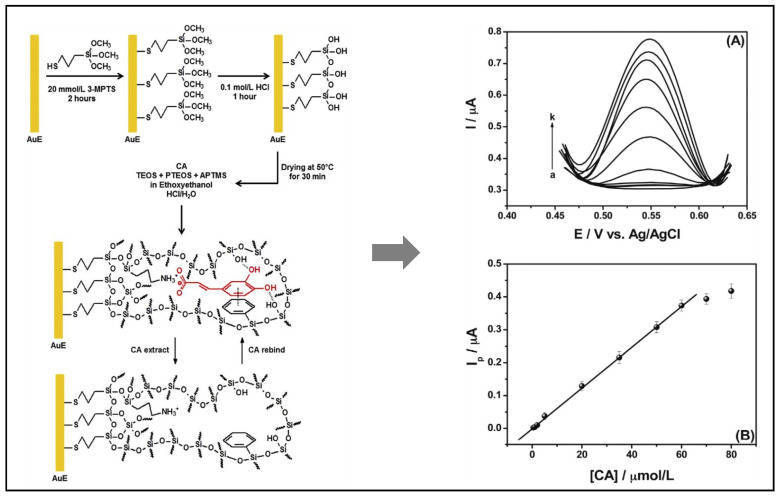
On the left: schematic representation of MIS/AuE, reproduced from [242] with the permission of Elsevier. On the right: (**A**) Differential pulse voltammograms for CA electrooxidation at different concentrations: (a) 0, (b) 0.500, (c) 1.00, (d) 2.00, (e) 5.00, (f) 20.0, (g) 35.0, (h) 50.0, (i) 60.0, (j) 70.0 and (k) 80.0 µmol/L. (**B**) Calibration plot, Ip/µA = 0.00619 (± 1.92 × 10^−4^) [CA]/µmol/L −0.00125 (± 4.36 × 10^−4^). Incubation time: 25 min. Supporting electrolyte: 0.4 mol/L sulfuric acid. Scan rate: 40 mV/s. Potential pulse amplitude: 70 mV, reproduced from [242] with the permission of Elsevier. 3-MPTS: (3-mercaptopropyl)trimethoxysilane, CA: caffeic acid, TEOS: tetraethoxysilane, PTEOS: phenyltriethoxysilane, APTMS: aminopropyltrimethoxysilane.

An electrochemical sensor using a gold electrode pre-modified with 3-mercaptopropyltrimethoxysilane and based on molecularly imprinted siloxanes was prepared for selective determination of caffeic acid in wines. The MIS film was prepared by sol-gel process, using the acid catalyzed hydrolysis and condensation of tetraethoxysilane (TEOS), phenyltriethoxysilane (PTEOS), and 3-aminopropyltrimethoxysilane (3-APTMS) in presence of caffeic acid as template molecule, then it was immobilized onto the modified electrode surface. DPV for CA oxidation were carried out at different concentrations. According to the author, the sensor was found to be highly selective toward the template, stable and repeatable. The sensor showed a linear current response to the target caffeic acid concentration in the range from 0.500 to 60.0 µmol/L, with a detection limit of 0.15 µmol/L (Figure 11) [242].

**Figure 12:**
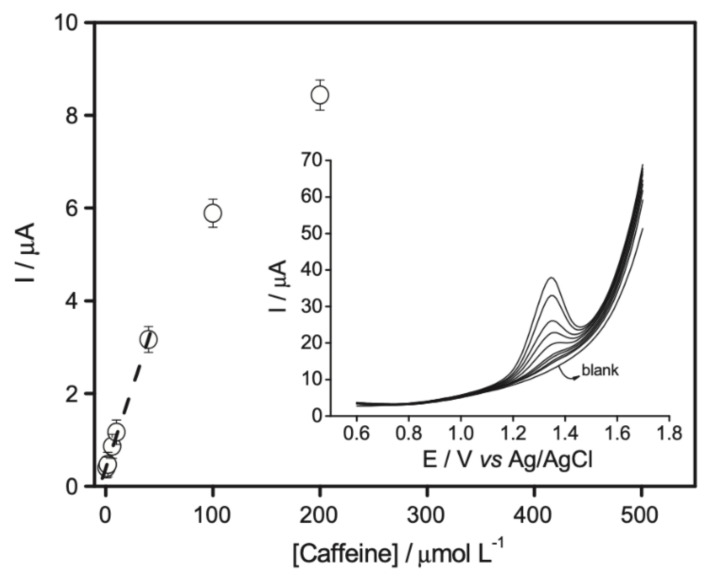
Current response (DPV) of the modified electrode recovered by siloxane film. Supporting electrolyte: 0.15 mol/L phosphoric acid. Incubation time: 15 min. DPV at 0.02 V/s. Reproduced from [243] with the permission of Elsevier.

A glassy carbon electrode modified with multiwall carbon nanotubes/vinyltrimethoxysilane recovered by siloxane film was developed for caffeine determination using DPV. Figure 12 shows a linear anodic peak current for caffeine concentration from 0.75 to 40 µmol/L^−1^ with high selectivity and sensitivity. The linear regression equation was ΔI/µA = 0.39 (± 0.04) + 0.07 (± 0.002) [caffeine]/µmol/L, *R*^2^ = 0.998. The detection limit was estimated to be 0.22 µmol/L [243].

**Figure 13:**
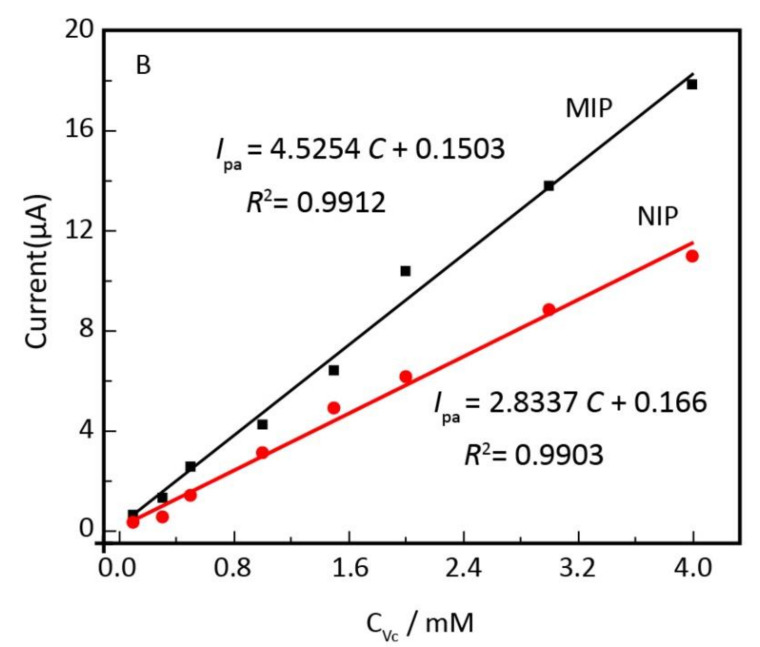
Linear relationship between anodic peak currents and the concentration of vitamin C (V_c_) for MIPs electrode and NIPs electrode, reproduced from [239] with the permission of Elsevier.

A molecularly imprinted electrochemical sensor based on polypyrrole (PPy) decorated with black phosphorene quantum dots (BPQDs) was prepared by electropolymerization onto poly 3,4-ethylenedioxythiophene (PEDOTNRs) for voltametric sensing of vitamin C. The peak currents recorded by DPV showed a linear proportionality on vitamin C concentrations ranging from 0.01 to 4 mM with a detection limit of 0.0033 mM (Figure 13). The prepared sensor demonstrated a good reproducibility, repeatability, stability, and selectivity for the electrochemical analysis of vitamin C [239].

**Figure 14:**
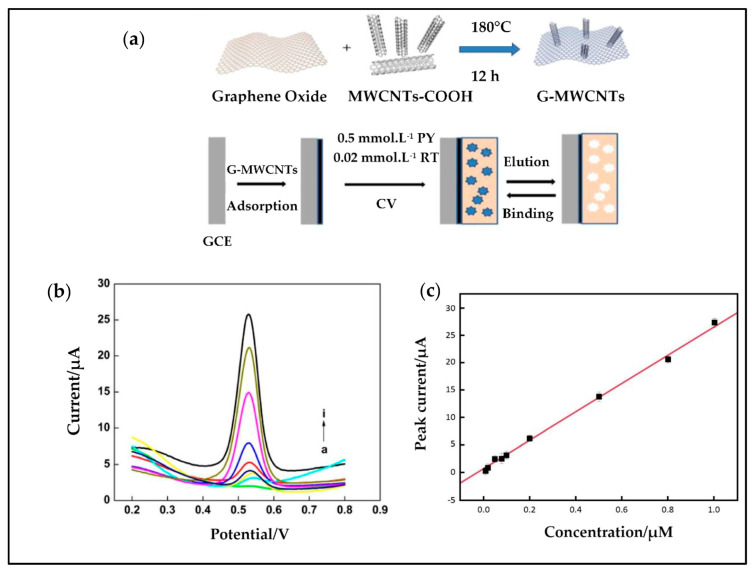
(**a**) Schematic construction of the MIP/G-MWCNTs/GCE electrode, G-MWCNTs: graphene-multiwall carbon nanotubes, GCE: glassy carbon electrode, PY: pyrrole, RT: rutin; (**b**) variation of DPVs with RT concentration; (**c**) linear relationship between peak current and RT concentration, reproduced from [244] with the permission of Elsevier.

Furthermore, a glassy carbon electrode was modified with molecularly imprinted polypyrrole-graphene-multiwalled carbon nanotubes composite film and used for rutin sensing (Figure 14a) and showed a proper increase of the peak current with increasing rutin concentrations (Figure 14b,c) where a linear relationship in the range of 0.01–1.0 µmol/L with a regression equation of ip(μA) = 26.18c (μmol/L) + 0.6308 (*R*^2^ = 0.997) was obtained [244].

A glassy carbon electrode was modified with molecularly imprinted polymer based on polypyrrole with incorporated graphene oxide for electrochemical determination of quercetin. Once the graphene oxide/glassy carbon was fabricated, an electropolymerization was carried out in a solution containing pyrrole, quercetin, and H_2_SO_4_. Cyclic voltametric experiments were performed on the modified electrode and oxidation peak current of quercetin was regressed with the concentration in the range from 6.0 × 10^−7^ to 1.5 × 10^−5^ mol/L (*R*^2^ = 0.997) with a detection limit of 4.8 × 10^−8^ mol/L. This electrode showed good stability and reproducibility [245].

Although we are actually more interested in natural antioxidants, an electrochemical sensor was prepared for tert-butylhydroquinone (TBHQ) recognition, a synthetic phenolic antioxidant that is extensively applied in food samples for its chemical stability, low cost, and availability; TBHQ-imprinted core–shell nanoparticles (TICSNs). TICNSs were fabricated in several steps. Silica spheres were synthesized and modified by (3-chloropropyl) trimethoxysilan, then by polyethylenimine, and polymerized to form the TICNs and polymerized to form the TICSNs with ethylene glycol dimethacrylate as the cross-linker. The resulting sensor was highly specific and selective. The linear range of the calibration curve was 0.1−50.0 mg kg^−1^ with the detection limit of 0.27 mg/kg [246].

**Figure 15:**
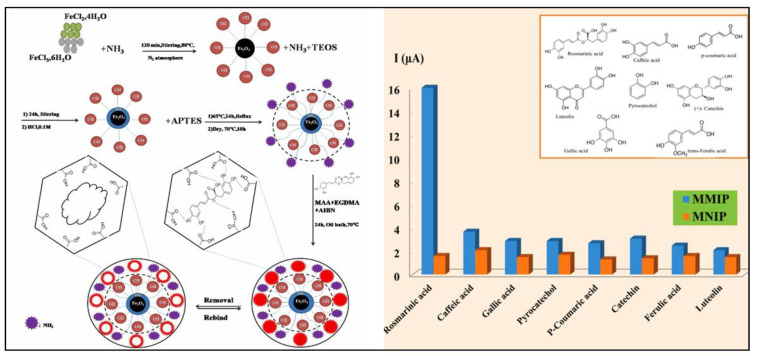
On the left: schematic representation of the applied approach for preparation of RA-MMIP, reproduced from [247] with the permission of Elsevier. On the right: Effect of other compounds on electrochemical determination of RA using MIP-CPE and NIP-CPE [247].

In addition, a modified carbon paste electrode (CPE) was designed based on magnetic functionalized molecularly imprinted polymer (MMIP) nanostructure for selective determination of rosmarinic acid (RA) in some plant extracts (*Salvia officinalis*, *Zataria multiflora*, *Mentha longifolia*, and *Rosmarinus officinalis*). The synthesis of MMIP was performed in four steps: (1) Iron oxide magnetite nanoparticles (Fe_3_O_4_ MNPs) were synthetized; (2) silica functionalized Fe_3_O_4_ MNPs (Fe_3_O_4_@SiO_2_) were synthesized and collected by a magnet, then washed and dried; (3) the surface of Fe_3_O_4_@SiO_2_ sample was modified using 3-amino propyltriethoxysilane (APTES); and (4) magnetic Fe_3_O_4_@SiO_2_@NH_2_ decorated with MIP was synthetized by surface polymerization. The CPE was modified with MMIP by mixing graphite powder, MMIP, and paraffin oil. All the steps are provided in Figure 15. The electrode behavior was studied with CV and DPV techniques. Two linear concentration ranges (0.1–100 μM and 100–500 μM) with a low detection limit (0.085 μM), and a good precision were obtained. The modified sensor showed good sensitivity and selectivity for the rosmarinic acid in the presence of other compounds (Figure 15) [247].

## 6. Conclusions

Given the major importance of antioxidants in the food industry, it would always be interesting to improve their evaluation methods.

While it is true that numerous techniques exist and have recently evolved, it should be noted that most of the classical ones suffer from a lack of selectivity, and among them techniques that are time consuming and costly, and others require large volumes of solvents. Consequently, it would be necessary to develop a technique for the determination of antioxidants that is fast, inexpensive, and has a good selectivity toward the desired compound and therefore to extend the applications that combine molecular imprinting, whether using MIP or MIS, with electrochemistry. This combination could lead to the development of an electrochemical sensor, consisting of an electrode on which a specific polymer will be deposited or directly synthesized on its surface, taking into consideration every critical step during the procedure, such as the choice of reagents for polymer synthesis, the synthesis technique, electrochemical methods and equipment, the choice of electrode, its modification, polymer deposition on the electrode, etc. Given the advantages of both techniques, this sensor could be very promising especially with the growing importance accorded to imprinted polymers with antioxidants, helping researchers and manufacturers to identify and detect one antioxidant at a time or a family of antioxidants, with high selectivity and specificity compared to other techniques, and in different media. For example wine, which is known to be very rich in antioxidants, or even olive oil, fruit juices, and many other food or cosmetic products, this sensor would be useful to identify the type and amount of antioxidants present in these products.

## Figures and Tables

**Table 1:** Amino acids susceptible to oxidation and their respective oxidation products, reproduced from [15] with the permission of Elsevier.

Amino Acid	Oxidation Products
**Cysteine** **Methionine** **Tyrosine** **Tryptophan** **Phenylalanine** **Valine, leucine** **Histidine** **Proline** **Threonine** **Arginine** **Lysine**	Disulfide, cystine Methione sulfoxide/sulphone Dityrosine, 3,4-dihydrophenylalanine (DOPA) Hydroxytryptophan,*N*-kynurenine, *N*-formylkynurenine, 3 hydroxylkynurenine Hydroxyphenylalanine, *o*-tyrosine, *m*-tyrosine Hydroxyperoxides 2-oxohistidine Hydroxyproline, glutamic semialdehyde pyrrolidinone 2-amino-3-ketobutyric acid Glutamic semialdehyde Hydroxylysine, 2-aminoadipic semialdehyde

**Table 2:** Some applications of CV and DPV techniques for the determination of antioxidants and antioxidant capacity in various analyzed media.

Antioxidants	Application Media	Working Electrode	Method	Linear Range (µM)	Detection Limit (µM)	References
**Polyphenols**	Black tea infusion	CNT electrode	CV	0.23–94	0.11	[86]
**Caffeic acid**	Red wine	SnO_2_-RGO/GCE	DPV	0.15–25	80.10^−3^	[87]
	Coffee	Au@α-Fe_2_O_3_@RGO/GCE	CV	19–1869	0.098	[88]
	Wine	F-GO/GCE	DPV	0.5–100	0.018	[89]
	Wine	Au/PdNPs-GRF	DPV	0.03–938.97	6 × 10^−3^	[90]
	Wine	RGO@PDA/GCE	DPV	5 × 10^−3^–450.55	1.2 × 10^−3^	[91]
**Gallic acid**	Tap water, tea and orange juice	SiO_2_ nanoparticles/CPE	DPV	8.0 × 10^−^ 1–1.0 × 10^−2^	2.5 × 10^−1^	[92]
	Wine	CS–fFe_2_O_3_–ERGO/GCE	DPV	1.0–1.0 × 10^6^	1.5 × 10^−1^	[93]
	Phosphate buffer solution	Zn-Al-NO_3_ layered double hydroxide film/GCE	DPV	4–600	1.6	[94]
**Gallic acid and total polyphenols**	Red and white wines	CNT modified carbon paste electrode	DPV	5.0 × 10^−1^–15	3.0 × 10^−1^	[95]
**Ascorbic acid**	Mixture of ascorbic acid, dopamine and uric acid	PG/GCE	CV	9.00–2314	6.45	[96]
	Aqueous solution	2,7-BFEFO/CPE	CV; DPV	50–2.65×10^3^; 9–3.5×10^3^	18; 4.2	[97]
	Fruit juices and wines	CPE; Pt strip electrode	DPV	70–20 × 10^3^; 310–20 × 10^3^	20; 87	[98]
	Flavored beverages	DNA/CPE	DPV	0.05–1.00	5 × 10^−4^	[99]
**Curcumin**	Human blood serum	NiCl_2_/GCE	DPV	10–600	0.109	[100]
	Spices	GCE	CV	9.9–1.07 × 10^2^	41	[101]
**Vanillic acid**	Artificial wine solutions	Graphite; carbon microspheres and CNT CPE	CV	10–400	2.85; 3.82; 4.13	[102]
**α-tocopherol; γ-tocopherol and δ-tocopherol**	Non-aqueous media	Pt electrode	DPV	2 × 10^−2^–10; 2.2 × 10^−2^–1.4; 2.21 × 10^−2^–31.1	1 × 10^−2^	[103]
**Quercetin**	*Rhizoma kaempferiae* and buds of *Styphnolobium japonicum* (L.) Schott	CTAB-cMWCNTs/MWCPE	CV	0.01–20	5.3 × 10^−3^	[104]

GCE: glassy carbon electrode, CNT: carbon nanotubes, SnO_2_-RGO: Tin(IV) oxide-reduced graphene oxide composite, F-GO: fluorine-doped graphene oxide, RGO@PDA: reduced graphene oxide and polydopamine composite, SiO_2_: silicon dioxide, CS: chitosan, fFe_2_O_3_: fishbone-shaped Fe_2_O_3_, ERGO: electrochemically reduced graphene oxide, BFEFO: 2,7-bis (ferrocenyl ethynyl) fluoren-9-one, Pt: platinum, CPE: carbon paste electrode, SPCE: screen-printed carbon electrode, PG: pristine graphene, NiCl_2_: nickel chloride, CTAB-cMWCNTs: cetyltrimethyl ammonium bromide-carboxylic multi-walled carbon nanotubes composite, MWCPE: multi-walled carbon paste electrode, CV: cyclic voltammetry, DPV: differential pulse voltammetry.

**Table 3:** Some MIPs applications with antioxidants.

Template	Application	Reference
**Tocopherols**	α-tocopherol delivery in gastrointestinal simulating fluids.	[151]
	Tocopherol recognition	[152]
**Quercetin**	Preconcentration and clean-up of catechins	[153]
	Extraction of anthocyanin from mangosteen pericarp	[154]
	Extraction of quercetin and kaempferol from the hydrolyzate of ginkgo leaves	[155]
	Separation of active inhibitors of epidermal growth factor receptor (EGRF) from Caragana Jubata	[156]
	solid-phase extraction for the sample pretreatment of natural products prior to HPLC analysis	[157]
**(+)-Catechin**	Extraction of catechins from tea extracts	[158]
	Retention of catechin	[159]
**Caffeic acid**	Separation and purification of chlorogenic acid	[143]
	Extraction of CA in commercial apple juice samples	[160]
	Selective extraction of polyphenols from olive mill waste waters	[161]
	Extraction of CA from fruits	[162]
	Separation and purification of the antioxidant compounds from mushrooms	[163]
**p-hydroxybenzoic acid**	Selective extraction of polyphenols from olive mill waste waters	[161]
**Resveratrol**	Selective recognition of resveratrol	[164]
